# Development and Internal Evaluation of an Interpretable AI-Based Composite Score for Psychosocial and Behavioral Screening in Dental Clinics Using a Mamdani Fuzzy Inference System

**DOI:** 10.3390/medicina62020412

**Published:** 2026-02-21

**Authors:** Alexandra Lavinia Vlad, Florin Sandu Blaga, Ioana Scrobota, Raluca Ortensia Cristina Iurcov, Gabriela Ciavoi, Anca Maria Fratila, Ioan Andrei Țig

**Affiliations:** 1 Doctoral School of Biomedical Sciences, Faculty of Medicine and Pharmacy, University of Oradea, 410087 Oradea, Romania; alvlad@uoradea.ro; 2 Industrial Engineering Department, Faculty of Management and Technological Engineering, University of Oradea, 1 Universității Street, 410087 Oradea, Romania; 3 Department of Dental Medicine, Faculty of Medicine and Pharmacy, University of Oradea, 410068 Oradea, Romania; gciavoi@uoradea.ro (G.C.); itig@uoradea.ro (I.A.Ț.); 4 Faculty of Medicine, Lucian Blaga University of Sibiu, 550169 Sibiu, Romania; anca.fratila@ulbsibiu.ro; 5 Military Clinical Emergency Hospital of Sibiu, 550024 Sibiu, Romania

**Keywords:** explainable AI, fuzzy inference system, Mamdani fuzzy, screening-oriented stratification, psychobehavioral composite score, dental workflow

## Abstract

*Background and Objectives:* Psychosocial symptoms and oral behaviors can complicate routine dental care, yet available screeners yield multiple separate scores. Explainable artificial intelligence offers a pragmatic way to integrate such multidomain measures into a single, auditable output that can support screening-oriented stratification and standardized documentation (non-diagnostic). Therefore, we aimed to develop an interpretable, deterministic Mamdani fuzzy inference system (FIS) integrating GAD-7, PHQ-9, and OBC-21 into a 0–10 psychobehavioral composite score (PCS) to support screening-oriented stratification and standardized documentation (non-diagnostic). *Materials and Methods:* Cross-sectional multicenter study in 18 private dental clinics in Romania (October 2024–March 2025; n = 460). A rule-based Mamdani Type-1 FIS was specified a priori (48 rules; triangular membership functions; centroid defuzzification) without supervised training. Internal evaluation assessed coherence across severity strata, robustness to predefined input perturbations (±1 point; ±5%) and membership-function variation (±10%), and benchmarking against linear composites (Z-mean; PCA PC1). *Results:* Median PCS was 2.30 (IQR 2.03–3.56). PCS correlated with GAD-7 (Spearman ρ = 0.886), PHQ-9 (ρ = 0.792), and OBC-21 (ρ = 0.687) (all *p* < 0.001), increased monotonically across anxiety and depression severity strata, and was higher in high OBC-21 risk. Robustness was excellent under input perturbations (ICC(3,1) = 0.983 for ±1 point; 0.992 for ±5%) and high under ±10% membership-function variation (ICC(3,1) = 0.959). Concordance with linear baselines was high (Spearman ρ = 0.956 for Z-mean; 0.955 for PCA PC1), with a small systematic nonlinearity at higher scores. *Conclusions:* PCS provides a fully auditable, rule-based integration of three patient-reported measures with coherent internal behavior and robustness to plausible measurement noise and specification changes. This study reports internal evaluation of a deterministic, rule-based aggregation; external clinical validation against independent outcomes is required before any clinical utility claims.

## 1. Introduction

General dental practice frequently encounters patients for whom psychological distress and oral behaviors may complicate routine care. Dental anxiety and broader anxiety-related problems are associated with avoidance patterns and may require adapted communication and pacing, strategies [1,2]. Recent syntheses also describe a bidirectional relationship between mental health and oral health, supporting the relevance of psychosocial screening in dental settings [3].

In the temporomandibular disorders (TMD) field, the biopsychosocial framework is embedded in the Diagnostic Criteria for Temporomandibular Disorders (DC/TMD). The DC/TMD emphasizes that psychosocial and behavioral factors (Axis II) can influence suffering, functioning, and clinical response, and may need early attention when planning care [4]. The framework also supports a stepped approach, from brief screening to comprehensive evaluation, and more recent recommendations extend the feasibility of short-form assessments to broader dental and primary care contexts [4,5].

Validated self-report instruments are available for screening anxiety, depressive symptoms, and oral parafunctional behaviors. However, routine use typically produces several separate scores that are not straightforward to integrate consistently into a single, reproducible summary for communication, documentation, and follow-up within dental workflows. An integration method should therefore support stratification while remaining transparent and auditable for clinical users [4,5,6].

Beyond interpretability, practical integration requirements are widely recognized as key determinants of whether medical AI systems are adopted and sustained in routine clinical workflows [7,8]. Rule-based fuzzy inference systems offer a transparent way to map numeric inputs into interpretable linguistic levels, apply explicit IF–THEN rules and generate a continuous output. Such systems can rep-resent graded severity and reduce reliance on rigid cutoffs while keeping the input–output logic inspectable [6,9,10]. In this context, three domains are particularly relevant for general screening in dental settings: anxiety symptoms, depressive symptoms, and oral behaviors [4,5]. The Generalized Anxiety Disorder-7 (GAD-7) and Patient Health Questionnaire-9 (PHQ-9) are widely used, validated measures for anxiety and depressive symptom severity [11,12,13,14]. The Oral Behaviors Checklist (OBC) quantifies oral behaviors, with published evidence supporting reliability and validity [15,16]. Despite their practicality, a gap remains between having multiple validated scores and having an explainable, single-number composite output designed for screening-oriented stratification in dentistry.

Therefore, the aim of this study was to develop an interpretable, deterministic Mamdani-type fuzzy inference system that integrates GAD-7, PHQ-9, and OBC total score into a single continuous 0–10 composite output intended for general psychosocial/behavioral stratification in dental settings. We further report an internal evaluation of mapping coherence across ordinal severity strata, agreement with transparent linear composite baselines, and robustness of the fuzzy score under predefined perturbations of inputs and membership-function specification. Full reproducibility is supported by providing the complete rule base and membership-function parameterization to facilitate exact reimplementation and to facilitate future external validation in independent samples and against clinically meaningful outcomes.

This composite output is intended as a non-diagnostic, screening-oriented summary to support communication and documentation alongside the original instrument scores. It does not replace clinical assessment or referral pathways.

## 2. Materials and Methods

### 2.1. Study Design

This study employed an observational, cross-sectional, multicenter, methodological design aimed at the development and internal validation of a fuzzy logic–based model for the integrative assessment of psychobehavioral composite score. Data were collected using standardized self-report instruments and processed as input variables within a fuzzy inference system.

The observational and cross-sectional approach was selected to allow the assessment of all variables at a single point in time, without intervention or randomization, ensuring that the model captured naturally occurring patterns within the study population. This design is appropriate for methodological research focused on model construction and internal validation rather than causal inference.

Data collection was conducted between October 2024 and March 2025 across multiple dental clinics located in different regions of the country. The multicenter structure was chosen to reduce single-site sampling bias and increase heterogeneity.

The methodological framework allowed the systematic use of patient-reported outcomes as inputs for the fuzzy inference system, supporting the development of an interpretable and robust computational model.

### 2.2. Participants and Sampling

During the study period, 483 patients were approached and assessed for eligibility across participating clinics. Twenty-three patients (4.8%) were excluded: 7 (1.4%) due to incomplete questionnaire data (item non-response), 1 (0.2%) due to a known severe psychiatric disorder, 12 (2.5%) due to acute dental/orofacial conditions, and 3 (0.6%) due to inability to comply with study procedures. No overlap between exclusion criteria was observed. The final analytic sample comprised 460 participants.

Inclusion criteria were age ≥ 18 years; attendance at one of the participating dental clinics between October 2024 and March 2025; ability to understand and complete the self-administered questionnaires in Romanian; and provision of written informed consent.

Exclusion criteria were incomplete questionnaire data (at least one unanswered item), known severe psychiatric disorders preventing reliable questionnaire completion, acute dental or orofacial conditions precluding reliable questionnaire completion (e.g., acute dental pain, abscess, severe inflammation, recent trauma, immediate postoperative status), and inability to comply with study procedures.

Eligible participants were enrolled consecutively across participating clinics. No priori sample size calculation was performed, as the study focused on model development and internal evaluation. Uncertainty in key internal evaluation estimates is reported using 95% confidence intervals where applicable.

### 2.3. Instruments

All participants included in the final analytic sample (N = 460) completed the Romanian versions of the Generalized Anxiety Disorder-7 (GAD-7) and Patient Health Questionnaire-9 (PHQ-9), and a Romanian version of the Oral Behavior Checklist-21 (OBC-21). The Romanian adaptation of the GAD-7 has been previously described psychometrically [17]. The PHQ-9 has undergone cross-cultural adaptation and validation in Romanian samples [18]. OBC-21 was rendered into Romanian by two bilingual experts with backgrounds in dentistry and psychology. The final version was agreed upon by consensus and slightly refined after pilot testing to ensure clarity and understanding [19].

Anxiety symptoms were assessed using the Generalized Anxiety Disorder-7 (GAD-7), a brief self-report instrument designed to screen for generalized anxiety disorder and to assess symptom severity in clinical and non-clinical populations. The GAD-7 consists of seven items referring to symptoms experienced over the previous two weeks, each rated on a four-point Likert scale from 0 (“not at all”) to 3 (“nearly every day”), yielding a total score ranging from 0 to 21, with higher scores indicating greater anxiety severity Cut-off scores of 5, 10, and 15 are typically used to indicate low, moderate, and severe anxiety levels, respectively. The GAD-7 has demonstrated robust psychometric properties, including high internal consistency and good validity, and has been further validated in large general population samples [11,12].

Depressive symptoms were assessed using the Patient Health Questionnaire-9 (PHQ-9), a self-report instrument for depression screening and symptom severity assessment. The PHQ-9 consists of nine items corresponding to DSM-IV criteria for a major depressive episode over the previous two weeks [13,14]. Each item is rated on a four-point Likert scale from 0 (“not at all”) to 3 (“nearly every day”), yielding a total score from 0 to 27, with higher scores indicating greater depressive symptom severity; cut-offs of 0–4, 5–9, 10–14, 15–19, and 20–27 were used to indicate minimal, low, moderate, moderately severe, and severe symptom levels, respectively [13,20]. The PHQ-9 has demonstrated robust psychometric properties, including good internal consistency and validity, and has been widely used across clinical and research settings [13,21].

To assess oral behaviors, the Oral Behaviors Checklist (OBC-21) was used. The OBC was developed within the RDC/TMD framework and is included in the DC/TMD Axis II assessment battery for both clinical and research applications [4]. The checklist comprises 21 items assessing the frequency of daytime and sleep-related oral behaviors over the previous month (e.g., clenching/grinding, object biting, cheek/lip biting). Each item is rated on a five-point ordinal scale from 0 (“never”) to 4 (“very often”), yielding a total score from 0 to 84, with higher scores indicating more frequent oral behaviors. For OBC-21, risk grades were defined as 0 (no risk), 1–24 (low risk), and 25–84 (high risk) [22]. Evidence supports the OBC’s reliability and validity for assessing oral overuse/parafunctional behaviors relevant to TMD and orofacial pain research [15].

### 2.4. Data Collection Procedure

Data were collected from participants included in the final analytic sample (N = 460) across 18 private dental clinics in Romania; analyses were performed on pooled data (no center-level modeling was performed). Questionnaires were completed either on paper or digitally using Google Forms, depending on availability and participant preference. The same questionnaire content and item order were used in both formats. The principal investigator coordinated data collection and ensured standardized instructions across sites; trained staff were available on-site to assist participants when needed. To minimize inter-operator variability across clinics, data collection was performed by local clinic staff under direct oversight of the principal investigator, who was present at all participating sites during data acquisition. Before data collection at each site, staff received a standardized on-site protocol briefing covering eligibility screening, in-formed-consent procedures, standardized participant instructions, and the boundaries of assistance (procedural clarification only, without interpreting questionnaire items). Identical questionnaire materials and administration order were used across centers, including consistent content across paper and digital formats. Data handling and quality-control procedures were applied uniformly across clinics, including completeness checks, double data entry with discrepancy resolution for paper forms, and consistency checks for digital exports.

Participants received written information regarding the study objectives, the voluntary nature of participation, and data confidentiality. Informed consent was obtained from all respondents: electronically via a dedicated checkbox for the digital form and by signature for the paper version. Questionnaire responses were collected without direct identifiers. Consent forms were stored separately from questionnaire data. Google Forms was configured not to collect email addresses or other identifying information. Data storage and processing complied with GDPR requirements.

The study involved no clinical interventions and was conducted in accordance with the Declaration of Helsinki. Ethical approval was obtained from the Research Ethics Committee of the Faculty of Medicine and Pharmacy, University of Oradea (approval number: CEFMF/2, dated 30 October 2023).

Data entry and quality control were standardized as follows: paper questionnaires were digitized via independent double data entry by two authors with discrepancy resolution against original forms, whereas digital questionnaires were exported directly and subjected to consistency checks. Analyses were conducted on de-identified questionnaire data, and PCS computation was fully deterministic given the input totals. Since no outcome labels or clinician-rated endpoints were used in this methodological study, conventional blinding to outcomes was not applicable; potential operator bias was minimized by self-administered standardized questionnaires, standardized participant instructions, and limiting staff involvement to procedural clarification without interpreting items.

### 2.5. Fuzzy Logic Model Development

A deterministic Mamdani fuzzy inference system (FIS) was developed to aggregate three self-report total scores (GAD-7, PHQ-9, and OBC-21) into a single continuous psychobehavioral composite score on a 0–10 scale (fuzzy score). The model was implemented in MATLAB R2024b Update 6 (24.2.0.2923080; 64-bit maci64) using the Fuzzy Logic Toolbox. The FIS was specified a priori (rule-based), with membership functions and the rule base defined analytically; no parameter fitting or supervised training against external criterion labels was performed. Defuzzification was performed using the centroid method.

#### 2.5.1. Inputs, Output, and Scoring Procedure

**Figure 1:**
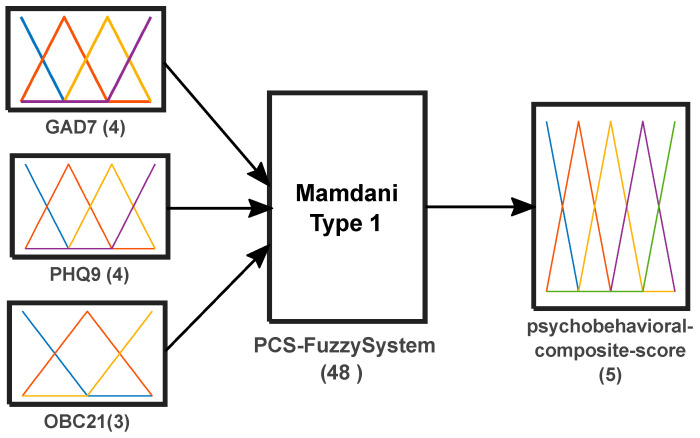
Architecture of the Mamdani-type fuzzy inference system (Type-1) used to compute the psychobehavioral fuzzy score.

The FIS used a Mamdani Type-1 architecture with three inputs and one output. Inputs were entered as raw total scores on their native scales: GAD-7 (0–21), PHQ-9 (0–27), and OBC-21 (0–84). GAD-7 and PHQ-9 were each represented by four linguistic terms (minimal, low, moderate, severe), whereas OBC-21 was represented by three linguistic terms (none, low, high). The output variable, psychobehavioral composite score (PCS), was defined on a continuous 0–10 universe and represented by five linguistic terms (none, low, moderate, moderately severe, severe); crisp scores were obtained using centroid defuzzification. We use the term ‘psychobehavioral’ to indicate that the composite integrates psychosocial symptoms (anxiety and depressive symptoms) and oral behaviors. For each participant, the composite score was computed via standard Mamdani inference (fuzzification, rule evaluation, aggregation, and centroid defuzzification). The FIS used the minimum operator for conjunction (AND), the maximum operator for disjunction (OR) and aggregation, the minimum operator for implication, and centroid defuzzification; the rule base comprised 48 rules. The overall FIS structure is illustrated in Figure 1.

#### 2.5.2. Membership Functions (MFs)

**Figure 2:**
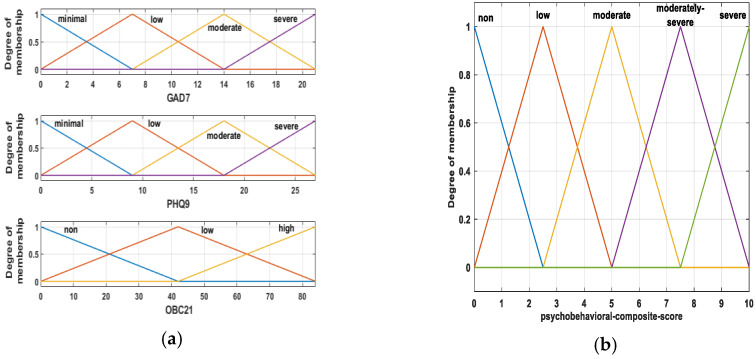
Triangular membership functions (trimf) used in the Mamdani Type-1 fuzzy inference system: (**a**) input variables (GAD-7, PHQ-9, OBC-21); (**b**) output variable (psychobehavioral composite score, 0–10).

All membership functions were triangular (trimf). Four linguistic terms were defined for GAD-7 and PHQ-9, three for OBC-21, and five for the output variable. The linguistic terms were GAD-7 (minimal, low, moderate, severe), PHQ-9 (minimal, low, moderate, severe), OBC-21 (none, low, high), and psychobehavioral composite score (none, low, moderate, moderately severe, severe). The triangular membership functions for the input variables and the output are shown in Figure 2. To avoid boundary effects, the outer MF supports were extended slightly beyond the theoretical score range, while inputs were still provided as raw totals on their native scales. In the FIS, PHQ-9 was fuzzified using four linguistic terms to maintain a parsimonious rule base. For descriptive reporting, PHQ-9 scores were additionally categorized using conventional five-level cut-offs; this categorization was independent of the FIS fuzzification. Input membership functions were anchored to conventional severity cut-offs (GAD-7: 5/10/15; PHQ-9: 5/10/15/20) with overlapping supports to allow graded transitions. OBC-21 linguistic levels were aligned to the predefined risk bands used in this study (0; 1–24; 25–84).

MF parameters and cut-offs were specified a priori for interpretability and were not optimized on this dataset or against any outcome. Also, MF parameters were not tuned to maximize agreement with any linear baseline or to fit the present dataset; they were anchored to conventional severity cut-offs (for GAD-7/PHQ-9) and predefined OBC-21 risk bands, and their impact was assessed via predefined ±10% perturbations (robust-ness analysis).

#### 2.5.3. Rule Base Construction and Specification

The rule base comprised 48 rules, corresponding to the full factorial combination of linguistic levels (4 GAD-7 levels × 4 PHQ-9 levels × 3 OBC-21 levels). The final rule list was explicitly specified in linguistic form (Table S1) and implemented identically in the MATLAB FIS. Rules were constructed systematically using an operational numeric-coding procedure. Each linguistic level was temporarily mapped to an increasing integer (GAD-7: 0–3; PHQ-9: 0–3; OBC-21: 0–2), and for each unique input combination a summed index was computed (Score = A + B + C). The summed index was then mapped to an output linguistic label using predefined score intervals, after which rules were expressed in standard IF–THEN linguistic form. This coding was used only to derive the rule consequents; fuzzy computation remained fully defined by real-valued inputs, membership functions, and the final linguistic rule base. All rules used unit weight (1.0) and a conjunctive connection (AND). The complete rule base is provided to support exact reimplementation without ambiguity.

#### 2.5.4. Inference Operators and Defuzzification

**Figure 3:**
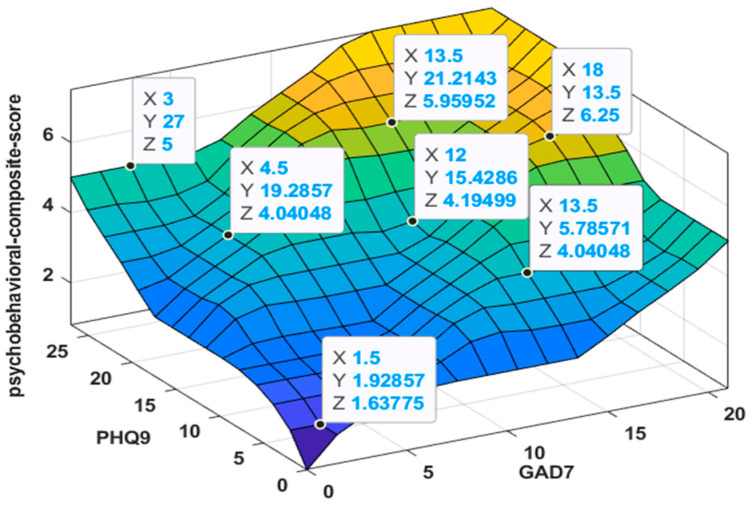
Response surface of the Mamdani Type-1 fuzzy inference system output (psychobehavioral composite score; 0–10) as a function of GAD-7 and PHQ-9, with OBC-21 held constant at 42 (midpoint of the 0–84 range).

Inference settings were fixed as follows: AND = min, OR = max, implication = min, aggregation = max, and defuzzification = centroid. The resulting input-output mapping is illustrated in Figure 3 as a response surface (GAD-7 and PHQ-9 on the axes, with OBC-21 held constant at 42). These settings, together with the MF parameterization and full rule base, fully determine the mapping from (GAD-7, PHQ-9, OBC-21) to the psychobehavioral composite score and allow exact reproduction of the scoring procedure.

### 2.6. Data Analysis

Primary statistical analyses were performed using IBM SPSS Statistics 25. Baseline computations (z-mean and PCA-derived PC1), linear-versus-quadratic model comparisons, and Bland–Altman analyses were implemented in Python 3.11.2 (Linux).

Continuous variables were assessed for distributional shape using the Shapiro–Wilk test and are summarized as mean (SD) or median (IQR), as appropriate. Between-group comparisons used the Mann–Whitney U test (two groups) or the Kruskal–Wallis H test (three or more groups), with Dunn–Bonferroni post hoc testing where applicable. Effect sizes were reported as r for Mann–Whitney tests and epsilon-squared (e2) for Kruskal–Wallis tests. Associations between quantitative variables were evaluated primarily using Spearman’s rho. Monotonic trends in the fuzzy score across ordinal severity categories were tested using the Jonckheere–Terpstra test.

Univariable and multivariable linear regression models were fitted with the fuzzy score as the dependent variable and GAD-7, PHQ-9, and OBC-21 as predictors. These models were used as descriptive benchmarks to summarize linear associations between the inputs and the derived fuzzy score (i.e., as an approximation of the fuzzy mapping), rather than for causal inference. Model summaries included beta coefficients, *p*-values, and adjusted R^2^; multicollinearity was assessed using variance inflation factors (VIF).

Robustness of the fuzzy score to input perturbations and membership-function (MF) specification was evaluated by recomputing the fuzzy score under three predefined perturbation scenarios. First, all three raw input totals (GAD-7, PHQ-9, and OBC-21) were perturbed simultaneously by an additive ±1 point change. Second, all three inputs were perturbed simultaneously by a proportional ±5% change. For both input-perturbation scenarios, values were capped to the allowable instrument ranges (GAD-7: 0–21; PHQ-9: 0–27; OBC-21: 0–84) prior to recomputing the fuzzy score. Third, MF parameters were perturbed by ±10% and the fuzzy score was recomputed. Agreement between the original and perturbed fuzzy scores was quantified using absolute differences and intraclass correlation coefficients (two-way mixed-effects design) with 95% confidence intervals.

To contextualize the fuzzy score, two linear composite baselines were derived from the same inputs: (i) an equal-weight baseline computed as the mean of standardized inputs (Z-mean), and (ii) a data-driven linear baseline defined as the first principal component (PC1) from PCA on standardized inputs. Concordance between the fuzzy score and each baseline was assessed using Spearman (primary) and Pearson (complementary) correlations, comparison of linear versus quadratic regression fit (R^2^) with nested model testing for the incremental quadratic term, and Bland–Altman analysis after linear calibration of baseline scores to the fuzzy score scale. The significance level was set at alpha = 0.05. We treated observations as independent and did not model center-level clustering; inferential *p*-values should therefore be interpreted cautiously.

## 3. Results

Table 1 presents the questionnaire-based characteristics of the analyzed participants (N = 460). Demographic characteristics are reported in Table S2; briefly, 364 (79.1%) participants were female, 326 (70.9%) resided in urban areas, and the most frequent age category was 20–29 (240/460, 52.2%). Most participants were classified as having low anxiety (39.3%), minimal depression (35.7%) or low depression (34.8%), and a low risk of oral parafunctional activity (60%). The median fuzzy score was 2.30 (IQR 2.03–3.56).

Results are presented in line with the five predefined study objectives, aimed at internally evaluating the fuzzy model and the resulting score. Specifically, we assessed internal coherence of the mapping (expected monotonic alignment with inputs and ordinal strata), numerical stability under minor variations in the input variables, severity-strata consistency (construct-consistent behavior across ordinal groups), the relative contribution of the input variables to the fuzzy score, and model robustness to variations in membership function parameters. As a sensitivity/contextualization analysis, we additionally benchmarked the fuzzy score against transparent linear composites derived from the same inputs.

### 3.1. Internal Coherence of the Mapping

**Figure 4:**
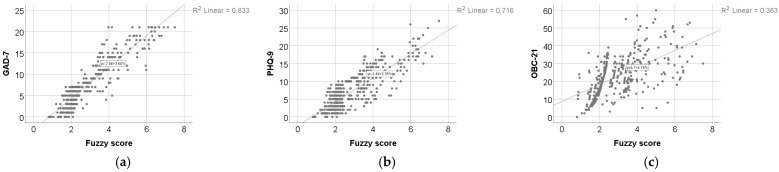
Associations between the fuzzy score and the three input questionnaire totals: (**a**) GAD-7 vs. fuzzy score; (**b**) PHQ-9 vs. fuzzy score; (**c**) OBC-21 vs. fuzzy score. Each panel shows the scatterplot with the corresponding linear fit and R^2^.

Figure 4 and Table 2 show significant positive associations between the psychometric test scores and the fuzzy score. Spearman’s rank correlation indicated a very strong correlation between the fuzzy score and GAD-7 (ρ = 0.886, *p* < 0.001), a strong correlation with PHQ-9 (ρ = 0.792, *p* < 0.001), and a moderate-to-strong correlation with OBC-21 (ρ = 0.687, *p* < 0.001), indicating that higher GAD-7, PHQ-9, and OBC-21 scores were associated with higher fuzzy scores.

### 3.2. Numerical Stability

Table 3 summarizes the stability of the fuzzy score under small perturbations of the input scores (±1 point and ±5%). All three inputs were perturbed simultaneously (±1 point and ±5%), with capping to valid score ranges, prior to recomputing fuzzy scores. Compared with the original fuzzy score (median 2.30 [IQR 2.03–3.56]), the modified models yielded small absolute differences. For the ±1-point perturbation, the median absolute difference between modified and original fuzzy scores was 0.187 (IQR 0.045–0.345) for +1 point and 0.159 (IQR 0.042–0.315) for −1 point, with 90th percentiles of 0.572 and 0.505, respectively. For the ±5% perturbation, median absolute differences were 0.073 (IQR 0.031–0.198) for +5% and 0.039 (IQR 0.031–0.166) for −5%, with 90th percentiles of 0.287 and 0.258, respectively. Agreement between original and modified fuzzy scores was excellent, with ICC(3,1) = 0.983 (95% CI 0.980–0.986) for the ±1-point condition and ICC(3,1) = 0.992 (95% CI 0.990–0.993) for the ±5% condition (both *p* < 0.001).

### 3.3. Severity-Strata Consistency

**Figure 5:**
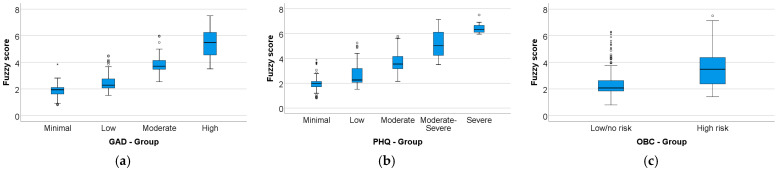
Fuzzy score distribution across severity/risk categories of the input measures: (**a**) GAD-7 severity groups; (**b**) PHQ-9 severity groups; (**c**) OBC-21 risk groups. Boxplots show median and interquartile range; whiskers represent the data spread according to the standard boxplot convention; points indicate outliers.

The fuzzy score was compared across GAD-7, PHQ-9, and OBC-21 severity groups (Figure 5; Table 4). The fuzzy score differed significantly across GAD-7 groups (Kruskal–Wallis, *p* < 0.001; ε^2^ = 0.716) and PHQ-9 groups (Kruskal–Wallis, *p* < 0.001; ε^2^ = 0.595), and increased monotonically with higher anxiety and depression severity (Jonckheere–Terpstra, both *p* < 0.001). Post hoc Dunn–Bonferroni analyses indicated significant increases in fuzzy score across the GAD-7 severity spectrum, with median values rising from 1.94 (minimal) to 2.29 (low), 3.71 (moderate), and 5.49 (high). For PHQ-9, median fuzzy scores increased from 1.98 (minimal) to 2.26 (low), 3.55 (moderate), 5.02 (moderate–severe), and 6.32 (severe); however, differences among the moderate and higher severity categories were not statistically significant after post hoc correction (*p* ≥ 0.062). Additionally, participants with high OBC-21 risk had higher fuzzy scores than those with low/no risk (Mann–Whitney U, *p* < 0.001; r = 0.563), with medians of 3.48 vs. 2.08.

### 3.4. Independent Contributions of Input Variables

Table 5 summarize univariable and multivariable linear regression models describing associations with the fuzzy score from GAD-7, PHQ-9, and OBC-21. In univariable models, all three scores were significant predictors (all *p* < 0.001). In the multivariable model, GAD-7 (B = 0.143, 95% CI 0.133–0.152; *p* < 0.001), PHQ-9 (B = 0.083, 95% CI 0.073–0.092; *p* < 0.001), and OBC-21 (B = 0.026, 95% CI 0.023–0.030; *p* < 0.001) remained independently associated with the fuzzy score. Model fit was high (adjusted R^2^ = 0.927; F(3,456) = 1950.232; *p* < 0.001). Diagnostics supported acceptable multicollinearity (VIF 2.401/2.233/1.274 for GAD-7/PHQ-9/OBC-21) and independence of errors (Durbin–Watson = 1.839). These models were used as descriptive benchmarks of linear contributions to the derived fuzzy score, rather than for causal inference.

### 3.5. Robustness to Fuzzy Parameter Variation

Table 6 summarizes the robustness of the fuzzy model to variations in membership function (MF) parameters (±10%). Compared with the original fuzzy score (median 2.30 [IQR 2.03–3.56]), modifying MF parameters produced small absolute differences in the resulting fuzzy scores. The median absolute difference was 0.145 (IQR 0.051–0.301) for −10% MF and 0.205 (IQR 0.083–0.451) for +10% MF, with 90th percentiles of 0.529 and 0.640, respectively. Agreement between the original and modified fuzzy scores remained high (ICC(3,1) = 0.959, 95% CI 0.952–0.968; *p* < 0.001), supporting robustness of the model output to MF parameter variation.

### 3.6. Sensitivity Analysis: Comparison with Linear Composite Baselines

**Figure 6:**
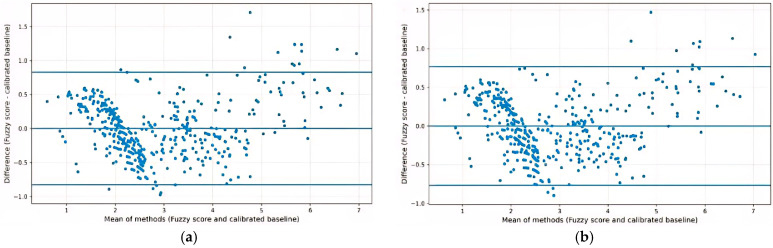
Bland–Altman plots comparing the fuzzy score with calibrated linear baseline composites: (**a**) Z-mean (equal-weight z-score composite); (**b**) PCA-derived PC1 baseline. Differences (fuzzy score − calibrated baseline) are plotted against the mean of methods; horizontal lines indicate the mean difference and the 95% limits of agreement.

Because all compared methods aggregate the same three monotonic inputs, high concordance with linear composites is expected and does not by itself indicate superiority of the fuzzy approach. The fuzzy score showed high concordance with linear composite baselines derived from the same inputs. Compared with the equal-weight z-score composite (Z-mean), concordance was high (Spearman rho = 0.956; Pearson r = 0.946). A linear model explained R^2^ = 0.896 of the variance in the fuzzy score, while adding a quadratic term increased fit to R^2^ = 0.924 (nested model comparison, *p* < 0.001), indicating a small localized deviation from a purely linear composite, most evident at higher score levels; this should be interpreted as a property of the predefined rule/MF mapping rather than as evidence of a superior data-driven structure. Bland–Altman analysis, performed after linear calibration of Z-mean to the 0–10 fuzzy score scale (baseline_cal = a + b*baseline), showed good agreement (95% limits of agreement approximately −0.828 to +0.828), with a modest proportional bias at higher score levels (slope = 0.057, *p* < 0.001) (Figure 6). Because Bland–Altman was performed after calibration to align scales, the analysis reflects residual disagreement rather than raw between-scale differences. After calibration, the median absolute difference was 0.294 (P95 = 0.808).

In sensitivity analyses using a PCA-derived baseline (PC1), results were similar (Spearman rho = 0.955; Pearson r = 0.954; R^2^ linear = 0.910; R^2^ quadratic = 0.939; 95% limits of agreement approximately −0.767 to +0.767). Z-mean was considered the primary linear comparator due to its transparency, while PCA (PC1) was used as a sensitivity analysis (Table 7).

## 4. Discussion

A persistent methodological challenge in psychosocial assessment is balancing integration with interpretability: composite indices may oversimplify relationships between constructs, whereas more complex models often reduce transparency and clinical acceptability. Fuzzy logic offers an intermediate solution by combining variables through explicit “if–then” rules, thereby preserving closer correspondence to clinical reasoning [23]. In this multicenter study, we developed an interpretable composite score that integrates anxiety (GAD-7), depressive symptoms (PHQ-9), and oral behavioral risk (OBC-21) into a single indicator of psychobehavioral vulnerability relevant to dental settings. The main message is that the resulting score exhibits construct-consistent behavior and maintains stability under minor fluctuations in input totals, providing evidence of internal coherence and stability. This approach may reduce fragmentation in the interpretation of the three instruments and support clinical stratification without substituting for diagnostic assessment. The next necessary step is external validation and evaluation of predictive utility against clinically meaningful outcomes.

Prior clinical evidence supports a clustering of emotional distress with stress-related oral behaviors. Vrbanović et al. reported positive associations between Oral Behaviours Checklist (OBC) frequency and both anxiety and depressive symptoms, alongside a strong interrelationship between anxiety and depression, and identified anxiety as a key predictor of oral behavior frequency in multivariable models [24]. Consistent with this pattern, in our multicenter dental sample, the proposed fuzzy composite score correlated positively with GAD-7, PHQ-9, and OBC-21, indicating that higher emotional symptom burden and more frequent oral behaviors align with higher integrated psychobehavioral vulnerability.

Importantly, the composite score was positively aligned with all three domains, supporting its ability to summarize co-occurring emotional and behavioral vulnerability rather than reflecting a single questionnaire alone. Taken together, these convergent associations support mapping coherence of the predefined aggregation, indicating that the rule base preserves the expected direction of severity across domains.

Building on this evidence, the fuzzy composite score showed significant positive monotonic associations with each of its three source domains (Table 2; Figure 1, Figure 2 and Figure 3). The relationship was most pronounced for anxiety, followed by depressive symptoms, while the association with oral behavioral risk was comparatively smaller yet consistent. This pattern suggests that the integrated output primarily tracks emotional symptom burden while retaining sensitivity to behavioral vulnerability that is clinically relevant in dental settings. However, because the score is derived from the same questionnaire totals, these monotonic patterns should be interpreted primarily as evidence of mapping coherence, indicating that the rule base and membership functions preserve the expected direction of severity across domains. This supports the fuzzy score as an internally coherent integrative index for stratification rather than as evidence of external criterion validity.

This ordering is plausible in a dental setting where acute psychological distress may co-occur with parafunctional behaviors [24], while self-reported oral behaviors may be more affected by recall bias, situational factors, and varying awareness [25,26]. Therefore, the comparatively smaller contribution of OBC-21 should not be interpreted as lack of relevance, but rather as reflecting the broader and potentially noisier behavioral construct relative to symptom scales [25].

Beyond domain alignment, the stability and robustness analyses address a practical property of any composite score derived from self-report instruments: minor fluctuations in questionnaire totals are common and should not produce disproportionate shifts in an integrative indicator [27]. In our data, small perturbations in input totals and modest membership-function parameter changes led to only limited changes in the fuzzy score, supporting controlled behavior of the aggregation under realistic measurement noise. This is particularly relevant if the score is used for stratification or triage in dental settings, where a robust composite may facilitate consistent communication and follow-up decisions. Importantly, this robustness should be interpreted as internal reliability of the mapping rather than as evidence of external predictive validity.

In benchmarking analyses, the fuzzy score was highly concordant with both Z-mean and PCA-based linear composites, supporting internal coherence of the aggregation and preservation of the ordering implied by the input scales. However, the better fit of a quadratic compared with a linear mapping and the Bland–Altman results indicate a small but systematic nonlinear deviation from a purely linear composite, more evident at higher score levels [28]. These findings support the fuzzy model as an interpretable, rule-based aggregation with controlled nonlinearity, without implying clinical superiority in the absence of an external criterion.

Prior work has applied Mamdani-type fuzzy inference systems in dentistry to integrate imprecise or heterogeneous inputs into interpretable risk estimates or treatment-oriented recommendations, typically using expert-defined rules and evaluating agreement against expert judgment or reference decisions [29]. Fuzzy logic has also been used for questionnaire-based mental-health severity staging and alternative scoring approaches, aiming to produce graded outputs rather than relying only on hard cut-offs [30]. Mago et al. proposed a fuzzy logic–based expert system for mobile-tooth management that maps imprecise sign–symptom inputs to suggested treatment options and evaluated its agreement with dentists’ decisions [31]. Several researchers [32] illustrate how a Mamdani fuzzy rule-based approach can be used to handle uncertainty in psychometric measurement and to generate graded outputs from questionnaire totals. Specifically, they developed two single-input/single-output fuzzy models to convert raw neuroticism and extraversion scores (collected using the Maudsley Personality Inventory) into standard scores, and then used these standard scores in a third fuzzy rule-based model to estimate participants’ anxiety levels based on the Sinha’s Comprehensive Anxiety Test. Model performance was evaluated using mean absolute percentage error (MAPE) and paired two-tailed t-tests, supporting the idea that deterministic, rule-based fuzzy mappings can provide an interpretable alternative to hard thresholds when translating raw questionnaire scores into severity-like constructs [32]. In line with this literature, the present PCS is a deterministic, rule-based mapping anchored to validated severity regions of the underlying instruments, designed primarily for transparency and auditability. However, unlike studies that report diagnostic accuracy or decision-level performance against external labels, our evaluation is restricted to internal characterization (monotonicity across severity strata, robustness to plausible perturbations, and benchmarking against transparent linear composites), because no external criterion or outcome-linked validation dataset was available.

Given the strong concordance observed, a simple linear composite can be an appropriate choice for many use cases. The rationale for the fuzzy approach in this work is not improved correlation but transparency and auditability of a predefined integration logic with graded transitions around familiar severity regions, which can be reviewed and locally refined without retraining.

From a practical standpoint, PCS computation requires a computational implementation; however, it does not require clinicians to be familiar with fuzzy logic. The mapping is deterministic and can be delivered through a simple calculator interface (e.g., embedded in an electronic form), so the end user sees only the PCS and its interpretive grade alongside the original instrument scores. Following external validation, we intend to provide an accessible web-based calculator and/or an open-source implementation to support reproducible computation.

The proposed fuzzy score can be seen as a pragmatic way to operationalize biopsychosocial screening in dental practices: it compresses anxiety (GAD-7), depressive symptoms (PHQ-9), and oral-behavior risk (OBC-21) into a single, auditable indicator that can be computed chairside. Because the mapping is explicitly rule-based, the contribution of each domain remains transparent and can be reviewed, refined, and locally calibrated, which may improve clinical acceptability compared with opaque predictive models. This positioning is consistent with DC/TMD recommendations that emphasize structured psychosocial assessment (including PHQ-9 and GAD-7) as part of dental evaluation, and with evidence suggesting that simplified scoring formats can be feasible in routine care [4,33]. In practical terms, an interpretable integrative score could support consistent communication and follow-up pathways (e.g., identifying patients who may benefit from additional psychosocial screening, behavioral counseling regarding parafunctional habits, or referral when indicated), without substituting for diagnosis or implying clinical superiority in the absence of an external criterion [4,29].

High concordance with linear composites is expected because all methods integrate the same three monotonic inputs. The added value of the FIS is not higher correlation, but an auditable rule-based nonlinearity that implements graded transitions around clinically familiar severity regions and avoids enforcing a single global linear trade-off between domains. In practice, this supports transparent review and local refinement of integration logic without retraining.

From a practical perspective, mapping the continuous PCS output to five interpretable grades provides a shared, auditable language for chairside screening and workflow adaptation. The proposed grades are intended to support proportional adjustments in communication, pacing, and follow-up intensity, rather than to mandate clinical decisions. Importantly, these grades represent model-defined categories (based on the fuzzy membership structure) and should not be interpreted as clinically validated thresholds.

Urgent dental needs should still be addressed. However, when marked distress, limited coping capacity, or reduced ability to engage in shared decision-making is suspected, elective or irreversible interventions may be deferred until the patient is better able to tolerate and adhere to the proposed plan. These proposed uses describe workflow adaptation and referral signposting, not diagnostic classification; the clinical impact and actionable thresholds require external validation.

Broader health-system context further supports the relevance of structured screening in dentistry. During the COVID-19 lockdown, when elective dental care was restricted, retrospective data from a Romanian dental emergency service showed an increased number of emergency presentations compared with pre- and post-lockdown periods, underscoring how constrained access can amplify workflow pressure and case complexity. In such contexts, an interpretable composite like PCS may help standardize documentation and support proportional adjustments in communication, pacing, and treatment sequencing, while preserving clinician judgment. Importantly, this study did not evaluate emergency settings, and any impact on service utilization would require external validation [34].

This work is inherently interdisciplinary, sitting at the interface of dentistry, behavioral sciences, and explainable AI. Dentistry provides the workflow context in which brief screening must be feasible; behavioral science provides validated constructs and instruments for anxiety, depressive symptoms, and oral behaviors; and explainable AI provides a transparent integration mechanism that can be inspected, audited, and refined. Uusing a deterministic rule-based fuzzy inference system, the proposed PCS is intended to support structured communication and proportional workflow adaptation while preserving clinician judgment. In this study, we report internal evaluation of the mapping and score properties only; external validation against clinically meaningful outcomes is required before any claims of clinical utility or deployment. This framing defines the next steps: external validation and prospective evaluation of performance and implementation within real-world, team-based care pathways.

### 4.1. Limitations

Several limitations should be considered when interpreting these findings. First, the study is cross-sectional; therefore, the results support internal coherence, stability, and robustness of the mapping, but do not allow causal inference or conclusions about temporal prediction. Second, all inputs are self-reported measures; consequently, reporting bias, response variability, and measurement error may affect questionnaire totals, and the fuzzy score necessarily inherits these sources of uncertainty, particularly for waking-state oral behaviors.

Third, although the rule base was specified systematically and the model is explicit and auditable through the specification of membership functions and system structure, the choice of the output universe (0–10), the number of linguistic levels (five), and the parametrization of output membership functions remain modeling decisions that can influence the score [23]. Robustness analyses under reasonable membership-function variations mitigate this risk but do not eliminate it. In addition, the five output levels represent model-defined categories rather than clinically validated cut-offs.

Fourth, model development and internal evaluation were performed within the same dataset. While we included stability analyses, robustness checks, and benchmarking against linear composites to contextualize the mapping, these procedures do not establish transportability to new populations and may yield optimistic agreement estimates compared with external samples. Although the sample was multicenter, the main analyses were performed at the individual level without explicit adjustment for site-level clustering (e.g., mixed-effects models or cluster-robust standard errors). If within-clinic correlation is present, uncertainty estimates may be biased downward, potentially inflating statistical significance. This limitation primarily affects inference (*p*-values and confidence intervals) rather than the descriptive patterns observed.

This study used a convenience, cross-sectional sample with an overrepresentation of younger participants and women, which limits generalizability and precludes causal inference. Although data were collected across multiple clinical sites, we did not apply mixed-effects modeling or clustering corrections; therefore, *p*-values may be optimistic if within-center correlation is present. Future work should evaluate center heterogeneity and use appropriate clustered/mixed-effects analyses, alongside external validation against clinically meaningful outcomes.

Finally, no external criterion (e.g., clinician-rated outcomes, confirmed diagnoses, or longitudinal endpoints) was available; therefore, we cannot claim clinical utility, diagnostic performance, or predictive validity, and we did not define clinically actionable thresholds. Also, alternative MF shapes, numbers of linguistic terms, and inference/aggregation configurations were not systematically compared in this study; such comparisons would require an external criterion and are left for future work.

Additionally, we did not evaluate usability or software integration; at present, PCS requires computational implementation (e.g., a simple calculator interface).

### 4.2. Future Directions

Future work will focus on prospective external validation of the psychobehavioral composite score (PCS) in independent dental samples, including assessment of transportability, calibration, and association with clinically meaningful outcomes (e.g., pain-related disability, care avoidance, treatment adherence, and referral decisions). These studies should also include explicit comparison with real-world clinical decisions and evaluation of added value relative to conventional aggregation baselines (e.g., standardized mean or PCA-based composites).

In a subsequent step, we plan to develop and evaluate an integrative transparent rule-based framework that combines the PCS (DC/TMD Axis II psychosocial/behavioral domain) with DC/TMD Axis I clinical findings and diagnoses, with the aim of supporting structured case formulation as a research framework. Any claims regarding decision support, triage, or diagnostic performance would require prospective validation against DC/TMD reference-standard assessments performed by calibrated examiners.

Following external validation, we also plan to provide an accessible web-based calculator and/or open-source implementation to enable reproducible computation and facilitate integration into electronic workflows, while avoiding actionable decision thresholds unless calibrated against clinically meaningful outcomes.

## 5. Conclusions

This study proposes an interpretable and reproducible fuzzy rule–based framework for aggregating psychometric scores, integrating GAD-7, PHQ-9, and OBC-21 into a single, auditable indicator. The resulting score showed construct-consistent behavior, with monotonicity across severity levels, stability under minor fluctuations in the input totals, and robustness to reasonable variations in membership-function parameters. Benchmarking against simple linear aggregation approaches (Z-mean and PCA-based composites) showed high concordance while also indicating a small, controlled deviation from a purely linear mapping at higher score levels. These findings document internal evaluation of a deterministic aggregation and define the next step: external validation in independent cohorts against clinically meaningful outcomes. Any assessment of predictive or triage utility requires prospective evaluation and is outside the scope of the present study.

## Figures and Tables

**Table 1:** Characteristics of the analyzed participants.

Parameter	Value
GAD-7 (Mean ± SD, Median (IQR))	7.63 ± 5.24, 7 (4–11)
GAD-7 groups (Nr., %)	
Minimal anxiety	138 (30%)
Low anxiety	181 (39.3%)
Moderate anxiety	85 (18.5%)
High anxiety	56 (12.2%)
PHQ-9 (Mean ± SD, Median (IQR))	7.24 ± 5.19, 6 (3–10)
PHQ-9 groups (Nr., %)	
Minimal depression	164 (35.7%)
Low depression	160 (34.8%)
Moderate depression	85 (18.5%)
Moderate-severe depression	41 (8.9%)
Severe depression	10 (2.2%)
OBC-21 (Mean ± SD, Median (IQR))	22.51 ± 10.36, 21 (15–29)
OBC-21 groups (Nr., %)	
No risk	1 (0.2%)
Low risk	276 (60%)
High risk	183 (39.8%)
Fuzzy score (Mean ± SD, Median (IQR))	2.891 ± 1.307, 2.30 (2.03–3.56)

**Table 2:** Correlations between analyzed scores and fuzzy score.

Correlation	Spearman Rho	*p*-Value
GAD-7 vs. Fuzzy score	0.886	<0.001
PHQ-9 vs. Fuzzy score	0.792	<0.001
OBC-21 vs. Fuzzy score	0.687	<0.001

**Table 3:** Evaluation of fuzzy model error behavior when changing input scores by ±1 point and ±5%.

Parameter	Fuzzy Score	Fuzzy Score (+1 Point)	Fuzzy Score (−1 Point)
Mean ± SD	2.89 ± 1.3	3.12 ± 1.36	2.68 ± 1.26
Median (IQR)	2.3 (2.03–3.56)	2.67 (2.07–3.83)	2.23 (1.93–3.36)
Differences—Modified fuzzy score—Original fuzzy score			
Difference	Mean ± SD	Median (IQR)	90th percentile
+1 Point—Orig.	0.236 ± 0.205	0.187 (0.045–0.345)	0.572
−1 Point—Orig.	0.207 ± 0.18	0.159 (0.042–0.315)	0.505
Parameter	Fuzzy score	Fuzzy score (+5% Score)	Fuzzy score (−5% Score)
Mean ± SD	2.89 ± 1.3	3.02 ± 1.39	2.78 ± 1.21
Median (IQR)	2.3 (2.03–3.56)	2.39 (2.06–3.70)	2.25 (1.99–3.46)
Differences—Modified fuzzy score—Original fuzzy score			
Difference	Mean ± SD	Median (IQR)	90th percentile
+5% Score—Orig.	0.129 ± 0.135	0.073 (0.031–0.198)	0.287
−5% Score—Orig.	0.105 ± 0.120	0.039 (0.031–0.166)	0.258

ICC(3,1) = 0.983 (95% C.I. = 0.980–0.986), F(459,918) = 177.303, *p* < 0.001. ICC(3,1) = 0.992 (95% C.I. = 0.990–0.993), F(459,918) = 355.283, *p* < 0.001.

**Table 4:** Comparison of the fuzzy score according to GAD-7/PHQ-9/OBC-21 groups.

Scale/Group	Mean ± SD	Median (IQR)	Mean Rank	Overall Test *p*	Effect Size
GAD-7					
Minimal	1.86 ± 0.4	1.94 (1.62–2.11)	102.10	<0.001	ε^2^= 0.716
Low	2.44 ± 0.57	2.29 (2.08–2.76)	208.64		
Moderate	3.86 ± 0.59	3.71 (3.47–4.15)	359.18		
High	5.41 ± 1.02	5.49 (4.51–6.25)	422.26		
PHQ-9					
Minimal	1.97 ± 0.5	1.98 (1.71–2.15)	123.02	<0.001	ε^2^= 0.595
Low	2.6 ± 0.78	2.26 (2.07–3.19)	219.74		
Moderate	3.73 ± 0.84	3.55 (3.15–4.16)	344.21		
Moderate-Severe	5.11 ± 1.06	5.02 (4.20–6.11)	413.35		
Severe	6.43 ± 0.48	6.32 (6.08–6.72)	449.15		
OBC-21					
Low/no risk	2.38 ± 0.99	2.08 (1.85–2.64)	169.61	<0.001	r = 0.563
High risk	3.66 ± 1.35	3.48 (2.37–4.4)	322.66		

Kruskal–Wallis H test (GAD-7 and PHQ-9), Mann–Whitney U test (OBC-21); effect sizes reported as epsilon-squared (ε^2^) or r.

**Table 5:** Univariable and multivariable linear regression models assessing associations with the fuzzy score.

Parameter	Univariable		Multivariable *	
	B (95% C.I.)	*p*	B (95% C.I.)	*p*
GAD-7	0.228 (0.218–0.237)	<0.001	0.143 (0.133–0.152)	<0.001
PHQ-9	0.213 (0.201–0.225)	<0.001	0.083 (0.073–0.092)	<0.001
OBC-21	0.076 (0.067–0.085)	<0.001	0.026 (0.023–0.030)	<0.001

* Multivariable linear regression model, Adjusted R^2^ = 0.927, F(3,456) = 1950.232, *p* < 0.001, Durbin-Watson score = 1.839, VIF: 2.401/2.233/1.274 (GAD-7/PHQ-9/OBC-21).

**Table 6:** Robustness analysis under ±10% membership function parameter variation.

Parameter	Fuzzy Score	Fuzzy Score (−10% MF)	Fuzzy Score (+10% MF)
Mean ± SD	2.89 ± 1.3	3.03 ± 1.19	2.76 ± 1.49
Median (IQR)	2.3 (2.03–3.56)	2.67 (2.08–3.59)	2.36 (1.79–3.49)
Differences—Modified fuzzy score—Original fuzzy score			
Difference	Mean ± SD	Median (IQR)	90th percentile
−10% MF—Orig.	0.207 ± 0.192	0.145 (0.051–0.301)	0.529
+10% MF—Orig.	0.281 ± 0.251	0.205 (0.083–0.451)	0.640

ICC(3,1) = 0.959 (95% C.I. = 0.952–0.968), F(459,918) = 70.788, *p* < 0.001.

**Table 7:** Comparison with linear composite baselines.

Baseline	Spearman Rho	Pearson r	R^2^ (Linear)	R^2^ (Quadratic)	LOA Low	LOA High	Median |diff|	P95 |diff|
Z-mean (equal-weight z-score composite)	0.956	0.946	0.896	0.924	−0.828	0.828	0.294	0.808
PCA PC1 (first principal component)	0.955	0.954	0.910	0.939	−0.767	0.767	0.284	0.734

LOA (limits of agreement) and |diff| metrics are computed from Bland–Altman analysis after linear calibration of each baseline to the fuzzy score scale; values are reported in fuzzy-score units.

## Data Availability

The original contributions presented in this study are included in the article. Further inquiries can be directed to the corresponding authors on reasonable request.

## Supplementary Materials

The following supporting information can be downloaded at: https://www.mdpi.com/article/10.3390/medicina62020412/s1, Table S1. Complete 48-rule Mamdani FIS rule list for PCS generation (inputs GAD-7, PHQ-9, OBC-21; output PCS); Table S2. Demographic characteristics of the analyzed participants (N = 460).

## Abbreviations

The following abbreviations are used in this manuscript:

AI-CDSS	Artificial Intelligence–Based Clinical Decision Support System
CI	Confidence Interval
DC/TMD	Diagnostic Criteria for Temporomandibular Disorders
DSM-IV	Diagnostic and Statistical Manual of Mental Disorders, Fourth Edition
FIS	Fuzzy Inference System
GDPR	General Data Protection Regulation
GAD-7	Generalized Anxiety Disorder-7
ICC	Intraclass Correlation Coefficient
IQR	Interquartile Range
MF	Membership Function
OBC-21	Oral Behaviors Checklist-21
PCA	Principal Component Analysis
PC1	First Principal Component
PCS	Psychobehavioral Composite Score
PHQ-9	Patient Health Questionnaire-9
RDC/TMD	Research Diagnostic Criteria for Temporomandibular Disorders
SD	Standard Deviation
TMD	Temporomandibular Disorders
