# Supplementary material for: Development and Internal Evaluation of an Interpretable AI-Based Composite Score for Psychosocial and Behavioral Screening in Dental Clinics Using a Mamdani Fuzzy Inference System
Source: Medicina (Kaunas). 2026 Feb 21;62(2):412. doi: 10.3390/medicina62020412 (PMC12943033; doi:10.3390/medicina62020412)
Supplement: Supplementary file 1 [file medicina-62-00412-s001.zip › Table S1. Complete 48-rule Mamdani FIS rule list for PCS generation (inputs GAD-7, PHQ-9, OBC-21; output PCS).pdf]

Table S1. Complete 48-rule Mamdani FIS rule list for PCS generation (inputs: GAD-7, PHQ-9, OBC-21; output: PCS)

1. If (GAD7 is minimal) and (PHQ9 is minimal) and (OBC is non) then (profil_psihocomportamental is non)
2. If (GAD7 is minimal) and (PHQ9 is minimal) and (OBC is low) then (profil_psihocomportamental is non)
3. If (GAD7 is minimal) and (PHQ9 is minimal) and (OBC is high) then (profil_psihocomportamental is low)
4. If (GAD7 is minimal) and (PHQ9 is low) and (OBC is non) then (profil_psihocomportamental is non)
5. If (GAD7 is minimal) and (PHQ9 is low) and (OBC is low) then (profil_psihocomportamental is low)
6. If (GAD7 is minimal) and (PHQ9 is low) and (OBC is high) then (profil_psihocomportamental is moderate)
7. If (GAD7 is minimal) and (PHQ9 is moderate) and (OBC is non) then (profil_psihocomportamental is low)
8. If (GAD7 is minimal) and (PHQ9 is moderate) and (OBC is low) then (profil_psihocomportamental is low)
9. If (GAD7 is minimal) and (PHQ9 is moderate) and (OBC is high) then (profil_psihocomportamental is moderate)
10. If (GAD7 is minimal) and (PHQ9 is severe) and (OBC is non) then (profil_psihocomportamental is low)
11. If (GAD7 is minimal) and (PHQ9 is severe) and (OBC is low) then (profil_psihocomportamental is moderate)
12. If (GAD7 is minimal) and (PHQ9 is severe) and (OBC is high) then (profil_psihocomportamental is moderately_severe)
13. If (GAD7 is low) and (PHQ9 is minimal) and (OBC is non) then (profil_psihocomportamental is non)
14. If (GAD7 is low) and (PHQ9 is minimal) and (OBC is low) then (profil_psihocomportamental is low)
15. If (GAD7 is low) and (PHQ9 is minimal) and (OBC is high) then (profil_psihocomportamental is moderate)
16. If (GAD7 is low) and (PHQ9 is low) and (OBC is non) then (profil_psihocomportamental is non)
17. If (GAD7 is low) and (PHQ9 is low) and (OBC is low) then (profil_psihocomportamental is low)
18. If (GAD7 is low) and (PHQ9 is low) and (OBC is high) then (profil_psihocomportamental is moderate)
19. If (GAD7 is low) and (PHQ9 is moderate) and (OBC is non) then (profil_psihocomportamental is low)
20. If (GAD7 is low) and (PHQ9 is moderate) and (OBC is low) then (profil_psihocomportamental is moderate)
21. If (GAD7 is low) and (PHQ9 is moderate) and (OBC is high) then (profil_psihocomportamental is moderately_severe)
22. If (GAD7 is low) and (PHQ9 is severe) and (OBC is non) then (profil_psihocomportamental is moderate)
23. If (GAD7 is low) and (PHQ9 is severe) and (OBC is low) then (profil_psihocomportamental is moderate)
24. If (GAD7 is low) and (PHQ9 is severe) and (OBC is high) then (profil_psihocomportamental is moderately_severe)
25. If (GAD7 is moderate) and (PHQ9 is minimal) and (OBC is non) then (profil_psihocomportamental is low)
26. If (GAD7 is moderate) and (PHQ9 is minimal) and (OBC is low) then (profil_psihocomportamental is low)
27. If (GAD7 is moderate) and (PHQ9 is minimal) and (OBC is high) then (profil_psihocomportamental is moderate)
28. If (GAD7 is moderate) and (PHQ9 is low) and (OBC is non) then (profil_psihocomportamental is low)
29. If (GAD7 is moderate) and (PHQ9 is low) and (OBC is low) then (profil_psihocomportamental is moderate)
30. If (GAD7 is moderate) and (PHQ9 is low) and (OBC is high) then (profil_psihocomportamental is moderately_severe)
31. If (GAD7 is moderate) and (PHQ9 is moderate) and (OBC is non) then (profil_psihocomportamental is moderate)
32. If (GAD7 is moderate) and (PHQ9 is moderate) and (OBC is low) then (profil_psihocomportamental is moderate)
33. If (GAD7 is moderate) and (PHQ9 is moderate) and (OBC is high) then (profil_psihocomportamental is moderately_severe)
34. If (GAD7 is moderate) and (PHQ9 is severe) and (OBC is non) then (profil_psihocomportamental is moderate)
35. If (GAD7 is moderate) and (PHQ9 is severe) and (OBC is low) then (profil_psihocomportamental is moderately_severe)
36. If (GAD7 is moderate) and (PHQ9 is severe) and (OBC is high) then (profil_psihocomportamental is severe)
37. If (GAD7 is severe) and (PHQ9 is minimal) and (OBC is non) then (profil_psihocomportamental is low)
38. If (GAD7 is severe) and (PHQ9 is minimal) and (OBC is low) then (profil_psihocomportamental is moderate)
39. If (GAD7 is severe) and (PHQ9 is minimal) and (OBC is high) then (profil_psihocomportamental is moderately_severe)
40. If (GAD7 is severe) and (PHQ9 is low) and (OBC is non) then (profil_psihocomportamental is moderate)
41. If (GAD7 is severe) and (PHQ9 is low) and (OBC is low) then (profil_psihocomportamental is moderate)
42. If (GAD7 is severe) and (PHQ9 is low) and (OBC is high) then (profil_psihocomportamental is moderately_severe)
43. If (GAD7 is severe) and (PHQ9 is moderate) and (OBC is non) then (profil_psihocomportamental is moderate)
44. If (GAD7 is severe) and (PHQ9 is moderate) and (OBC is low) then (profil_psihocomportamental is moderately_severe)
45. If (GAD7 is severe) and (PHQ9 is moderate) and (OBC is high) then (profil_psihocomportamental is severe)

46. If (GAD7 is severe) and (PHQ9 is severe) and (OBC is non) then (profil_psihocomportamental is moderately_severe)
47. If (GAD7 is severe) and (PHQ9 is severe) and (OBC is low) then (profil_psihocomportamental is moderately_severe)
48. If (GAD7 is severe) and (PHQ9 is severe) and (OBC is high) then (profil_psihocomportamental is severe)
