# Supplementary material for: Development and Internal Evaluation of an Interpretable AI-Based Composite Score for Psychosocial and Behavioral Screening in Dental Clinics Using a Mamdani Fuzzy Inference System
Source: Medicina (Kaunas). 2026 Feb 21;62(2):412. doi: 10.3390/medicina62020412 (PMC12943033; doi:10.3390/medicina62020412)
Supplement: Supplementary file 1 [file medicina-62-00412-s001.zip › Table S2. Demographic characteristics of the analyzed participants (N = 460).pdf]

Table S2. Demographic characteristics of the analyzed participants (N = 460)

Characteristic	Category	n	%
Sex	Female	364	79.1
	Male	96	20.9
Residence	Urban	326	70.9
	Rural	134	29.1
Age (years)	18	8	1.7
	20-29	240	52.2
	30-39	103	22.4
	40-49	52	11.3
	50-59	32	7.0
	60-69	18	3.9
	70-79	6	1.3
	>=80	1	0.2

Note: Percentages are calculated using N = 460.
